# Supplementary material for: Bridging self-directed learning and competency in interdisciplinary teaching among STEM lecturers: How psychological capital connects
Source: PLoS One. 2025 Jun 18;20(6):e0319305. doi: 10.1371/journal.pone.0319305 (PMC12176204; doi:10.1371/journal.pone.0319305)
Supplement: Attachment — Submitted filename: Editorial NoteDK- PONE-D-25-02654.pdf [file pone.0319305.s001.pdf]

Manuscript ID: PONE-D-25-02654

Bridging Self-directed Learning and Competency in Interdisciplinary Teaching Among STEM Lecturers: How Psychological Capital Connects

Editorial Note

This manuscript explores a rapidly emerging topic in higher education. It adds value to the existing literature by examining how Psychological Capital (PsyCap) mediates the relationship between Self-Directed Learning (SDL) and Competency in Interdisciplinary Teaching (CIT) among STEM lecturers. After thoroughly reviewing the manuscript and considering the reviewers' comments, I have provided my feedback and decision as below.

Abstract:

The abstract of this article outlines the core research idea. However, it is suggested to mention the key findings with some statistical results/data to strengthen the validity of the statement. It is also suggested to provide some limitations and future scope of this study thereby emphasizing the originality value statement.

Introduction/Literature Review:

Researchers are to be aware to include all the citations and their sources in the reference list. Several citations, such as (Liang & Li, 2023), (China Education Yearbook, 2023), (Pozo-Rico et al., 2023), (Jin, 2021), (Luthans, 2004), (Mc Clelland, 1973), (Knowles, 1975), (Zhao & Chen, 2024), (Greenhow et al., 2020), and many more., are not included in the reference/bibliography list, making it difficult to verify the sources or their relevance.

The constructs of each latent variable; SDL, CIT, and PsyCap, are well-identified, and the respective hypotheses are effectively developed, supporting the conceptual framework. While PsyCap's mediating role is well-defined, incorporating moderating factors like institutional support, teaching experience, or disciplinary variations could further contextualize the model. Additionally, integrating recent empirical evidence from diverse educational contexts beyond China would enhance the study's generalizability and impact on global STEM education.

Methodology, Results, and Conclusion

The PLS-SEM approach ensures robustness over CB-SEM (Also acknowledged by reviewer 1), however, reporting model fit indices (e.g., SRMR, NFI) would enhance model evaluation. Although VIF values confirm no multicollinearity (<10), the R^2 for PsyCap (0.362) is relatively low; using adjusted R^2 could better account for model complexity and improve predictive accuracy.

Though the PLS-SEM approach is well-applied, reflecting the robustness over CB-SEM, the model fit indices (e.g., SRMR, NFI) could be reported for a more comprehensive evaluation of the model's overall fit. Similarly, the VIF values (<10) confirm no multicollinearity concerns, but the R^2 value for PsyCap (0.362) explaining SDL is slightly lower. To overcome the issue, reporting adjusted R^2 could help account for potential model complexity and better reflect predictive accuracy.

As indicated by Reviewer 1, conduct the Structural Model Assessment with robustness checks, including Linearity (Quadratic effect), Heterogeneity (FIMIX PLS), and Endogeneity (Gaussian Copula). You can use the citation from the provided paper link: Technology in farming: Unleashing farmers' behavioral intention for the adoption of agriculture 5.0 (https://journals.plos.org/plosone/article?id=10.1371).

Similarly, as emphasized by reviewer 1, the study recognizes its contributions and suggests SDL programs and PsyCap training, but it does not offer concrete policy recommendations or implementation strategies, especially for universities. Further, in the conclusion section, it is advised to briefly discuss its limitations (such as contextual limits and generalizability) and potential future research directions.

After carefully reviewing the manuscript, considering the reviewers' feedback, and emphasizing the aforementioned issues, the manuscript is deemed suitable for future publication with minor revisions that meet the standards required for publishing in PLOS ONE.

Editorial Decision: **Minor Revision**
