# Supplementary material for: Bridging self-directed learning and competency in interdisciplinary teaching among STEM lecturers: How psychological capital connects
Source: PLoS One. 2025 Jun 18;20(6):e0319305. doi: 10.1371/journal.pone.0319305 (PMC12176204; doi:10.1371/journal.pone.0319305)
Supplement: Attachment — Submitted filename: Comments to Author.pdf [file pone.0319305.s002.pdf]

Dear Author/s,

Thank you for submitting your manuscript, *Bridging Self-directed Learning and Competency in Interdisciplinary Teaching Among STEM Lecturers: How Psychological Capital Connects*, to *PLOS ONE*. Your study addresses a timely and relevant issue in higher education and provides valuable insights into the role of Psychological Capital in supporting interdisciplinary teaching among STEM lecturers. However, a few revisions are suggested before the manuscript can proceed further:

Revisions Required:

Abstract:

- Include statistical data of the result while stating the key findings to bring rigor and validate the research claims.
- Briefly mention study limitations and future research directions to emphasize the originality of the study.

Introduction & Literature Review:

- Ensure that all citations are properly included in the reference list. Missing references such as (Liang & Li, 2023), (China Education Yearbook, 2023), (Jin, 2021), etc., should be included.
- Consider incorporating recent empirical studies beyond China to improve the study's global relevance and generalizability.
- Address potential moderating variables (e.g., institutional support, teaching experience, or disciplinary variations) to strengthen the conceptual framework.

Methodology & Results:

- While the PLS-SEM approach is well-applied, reporting model fit indices (e.g., SRMR, NFI) would improve the robustness of the model evaluation.
- The R^2 value for PsyCap (0.362) is relatively low. Consider reporting Adjusted R^2 to account for model complexity and improve predictive accuracy.
- As suggested by Reviewer 1, conduct robustness checks for the Structural Model Assessment [including: Linearity (Quadratic effect), Heterogeneity (FIMIX-PLS), and Endogeneity (Gaussian Copula)]. Fir this, refer to the recommended paper: Technology in Farming: Unleashing Farmers' Behavioral Intention for the Adoption of Agriculture 5.0

Conclusion:

- Discuss study limitations (e.g., contextual constraints, generalizability issues) more explicitly.
- Provide clearer policy recommendations for universities and educational institutions on implementing SDL and PsyCap programs.

Addressing the above points will significantly enhance the clarity, rigor, and impact of the study.

I appreciate your contributions to this field and look forward to receiving the revised manuscript.

Academic Editor

PLOS One
